# Diverse Physiological Functions and Regulatory Mechanisms for Signal-Transducing Small GTPases

**DOI:** 10.3390/ijms21197291

**Published:** 2020-10-02

**Authors:** Takaya Satoh

**Affiliations:** Laboratory of Cell Biology, Department of Biological Science, Graduate School of Science, Osaka Prefecture University, 1-1 Gakuen-cho, Naka-ku, Sakai, Osaka 599-8531, Japan; tkysato@b.s.osakafu-u.ac.jp; Tel.: +81-72-254-7650

Diverse GTPases act as signal transducing enzymes in a variety of organisms and cell types [1]. Signal-transducing GTPases are categorized into two groups: heterotrimeric and monomeric (small) GTPases. Small GTPases exist as either GDP-bound or GTP-bound forms and adopt significantly different conformations depending on whether GDP or GTP is bound. The primary function of small GTPases is to serve as molecular switches of intracellular signal transduction, and the on/off states of the switch are basically determined by its bound guanine nucleotide, GTP or GDP. In general, the GTP-bound conformation represents the “on” state, which is capable of binding and activating downstream effectors, whereas the GDP-bound conformation represents the “off” state, which shows a much lower affinity to the effectors.

Cycling between these on/off states is very slow in the absence of upstream stimulatory signals. The affinity of GDP and GTP for a small GTPase is generally very high, and the dissociation rates of these nucleotides are very low. Therefore, the transition from the “off” state to the “on” state is very slow. Additionally, the hydrolysis of bound GTP—namely, the transition from the “on” state to the “off” state—is very slow. These kinetic characteristics are very important to ensure that the switching is under the control of stimulatory proteins for respective reactions, guanine nucleotide exchange factors (GEFs), and GTPase-activating proteins (GAPs). In principle, the activation of GEFs and inactivation of GAPs increase the GTP-bound “on” conformations, whereas the inactivation of GEFs and the activation of GAPs increase the GDP-bound “off” conformations. Virtually all the upstream signals indeed modulate the balance between the on/off states by controlling the enzymatic activities, subcellular localization, and protein–protein interactions of GEFs and GAPs. Therefore, the understanding of the regulatory mechanisms of GEFs and GAPs is critical for the understanding of the regulatory mechanisms of their target small GTPases. Furthermore, the action of GEFs and GAPs is believed to be specific not only to their substrates (i.e., small GTPases), but also to the upstream signals and cell types.

In this Special Issue, several reviews and articles particularly focus on the physiological roles and regulatory mechanisms for GEFs and GAPs. Tanna et al. [2] reviewed the role of GAPs that act on Arf family small GTPases in the regulation of actin cytoskeletal rearrangements. Maier et al. [3] reported the identification of Trio as a GEF that is responsible for Rac1 hyperactivation in podocytes. Rac1 hyperactivation is known to cause podocyte injury and glomerulosclerosis, leading to nephrotic syndrome. Schmitz et al. [4] described a role for the dimeric GEF Dck1/Lmo1 on the translocation of Rho5 (an ortholog of mammalian Rac1) from the plasma membrane to mitochondria in response to glucose starvation in the yeast *Saccharomyces cerevisiae*. Ke et al. [5] investigated the role of the testis-specific protein TBC1D21 in mammalian spermiogenesis. They reported that TBC1D21, which was previously identified as a GAP for Rab, interacts with Rap1 and may act as a GAP for Rap1 during murine spermatogenesis. Niftullayev and Lamarche-Vane [6] summarized the role of the Rho family of small GTPases and its regulators—i.e., GEFs and GAPs—in axon guidance and neurological disorders.

Five families of signal-transducing small GTPases—i.e., Ras, Rho, Rab, Arf, and Ran families—have been identified in eukaryotes ranging from yeasts to mammals. In this Special Issue, four reviews deal with the functions of Ras family members. Jaśkiewicz et al. [7] described the diverse physiological functions of the Ras family member Rap1. Schöneborn et al. [8] gave an overview of the role of Ras and Rheb GTPases in regulating the regeneration of brain neurons. In addition, the authors discussed the magnetic guidance of re-growing axons as an approach to guide axonal growth by activating GTPase signaling. Kaiser [9] provided a review on the role of small GTPases in cyclic nucleotide signaling in pathogenesis induced by apicomplexan parasites and kinetoplastids. In this review, the regulation of the Ras family member Rap1 by cAMP via its GEF EPAC was highlighted. Abdelkarim et al. [10] summarized the functions of the hypervariable region of the Ras family member K-Ras4B. The understanding of the role of the unique hypervariable region of K-Ras4B in cell signaling will provide insights into its therapeutic targeting in cancer.

Reviews on Rho family GTPases and their target proteins are also included in this Special Issue. Zamboni et al. [11] described the role of Rho family small GTPases in the regulation of neuronal networking. Particularly, the dysregulation of these GTPases related to intellectual disability and therapeutic approaches were discussed. Ito et al. [12] summarized the function of the Rho effector rhotekin and its binding partners in neuronal tissues and cancer cells. Aspenström [13] addressed the involvement of Rho family GTPases in cancer. In particular, the intrinsic and modulated features of the GDP/GTP cycle of the Rho family members were highlighted. Durand-Onaylı et al. [14] summarized the involvement of Rac GTPases in the development and progression of hematological malignancies. The specific contribution of the deregulation of the Rac protein in hematologic malignancies was described in detail. Flentje et al. [15] summarized the role of Rho family small GTPases in the process of endothelium reconstruction after surgical treatments for cardiovascular disease. The process includes the migration and proliferation of endothelial cells, and a better understanding of the role for small GTPases in endothelial cell migration and proliferation may lead to the development of novel agents to treat vascular disease.

In four original research articles, the novel functions and regulatory mechanisms of Rho family small GTPases are clarified. Huang et al. [16] described that the Rho family small GTPase Cdc42 negatively regulates the polymerization of the GTP-binding cytoskeletal protein Septin12 and is involved in the terminal structure formation of sperm heads. Takenaka et al. [17] investigated the role of the Rho family small GTPase Rac1 in insulin-stimulated glucose uptake in adipocytes, demonstrating that Rac1 acts as a switch of insulin signaling downstream of the protein kinase Akt2. Sterk et al. [18] analyzed the role of the small GTPase Rho5, the *Saccharomyces cerevisiae* ortholog of human Rac1, in oxidative stress response. In particular, they focused on the function of the hypervariable region at the carboxyterminal end, including a unique 98 amino acid insertion. Many small GTPases, including Rho family members, localize at the plasma membrane and inner membranes through post-translational lipid modifications at the carboxy terminal portion. Aslam et al. [19] investigated the effect of the prenylation inhibition of Rho family GTPases on the endothelial cytoskeleton and barrier properties of endothelial cells. They demonstrated that the inhibition of geranylgeranylation in endothelial cells exerts a biphasic effect on the endothelial barrier properties.

Additionally, two reviews focus on the functions of specific members of Rab and Arf families. Osanai [20] described the role for the Rab family member Rab38 in lung function on the basis of phenotypes of *Rab38*-mutant rodents and patients with Hermansky–Pudlak syndrome. Van Acker et al. [21] gave an overview of the role of the the Arf family small GTPase Arf6 in innate immunity and host–pathogen interactions and its action mechanisms.

This Special Issue also contains four original research articles reporting the novel function of specific Rab GTPases and their regulation. Pupo et al. [22] investigated the role of the Rab family small GTPase Rab5, which is localized in the early endosomes, in the regulation of mitosis. Kinesin-2 was proposed to participate in the movement of Rab5-containing endosomes and their localization at the spindle, and the endosomal transport at the onset of mitosis was suggested to be required to control the timing of nuclear envelope breakdown. Hanadate et al. [23] identified and characterized the non-catalytic subunit of a lipid flippase as an interacting protein of the Rab family small GTPase Rab8A in the enteric protozoan parasite *Entamoeba histolytica*. This study demonstrated that Rab8A mediates the transport of the lipid flippase P4-ATPase from the ER to the plasma membrane. Kang et al. [24] studied the Rab family small GTPase Ypt1p, which exhibits a stress-driven structural and functional switch from a GTPase to a molecular chaperone, and mediates thermotolerance in *Saccharomyces cerevisiae*. Gambarte Tudela et al. [25] investigated the intracellular distributions and functions of Rab39a and Rab39b isoforms and showed different subcellular distributions and functions in lipid transport.

In addition to the canonical five families, GTPases with complex domain structures are also involved in intracellular signal transduction. Two reviews in this Special Issue focus on the structure and regulation of the Roco family GTPase LRRK2. Liao and Hoang [26] summarized the regulatory mechanisms of the new class of GTPases Roco. Aberrant activity in the human Roco protein LRRK2 is associated with the pathogenesis of Parkinson’s disease and, therefore, a therapeutic approach targeting this family of small GTPases may be important. Another review describing the mechanisms underlying the regulation of Roco family GTPases was given by Wauters et al. [27]. In this review, a novel hypothesis regarding the on/off cycle of the LRRK2 protein based on structural and biochemical analyses was proposed. Gijsbers et al. [28] reported the role of another GTPase in cell signaling. They described the interaction between the Shwachman–Bodian–Diamond syndrome (SBDS) protein and the GTPase elongation factor like-1. The SBDS protein acts as a GEF for elongation factor like-1, and all the tested mutations in the SBDS gene severely weakened the binding to elongation factor like-1, highlighting the importance of the interaction for their function.

Small GTPases serve as a key regulator of intracellular signaling in a wide variety of cell types, as described above. In many cases, defects in the function of small GTPases due to amino acid changes or decreased expression levels may cause various diseases depending on tissues and cell types. Therefore, not only small GTPases themselves but also their regulators and effectors are promising candidates for therapeutic targets. These points are important and addressed in virtually all the reviews and original research articles in this Special Issue.
